# Dynamic Coordination: How ERF Transcription Factors Coordinate Plant Development and Adaptive Stress Responses

**DOI:** 10.3390/biom16030466

**Published:** 2026-03-19

**Authors:** Mingcheng Wang, Panyue Du, Liyang Xi, Haifeng Lin, Shuqiao Zhang

**Affiliations:** 1 Institute for Advanced Study, Chengdu University, No. 2025 Chengluo Road, Chengdu 610106, China; 2 Sichuan-Xizang Medicinal Resource Breeding and Standardization Team, Engineering Research Center of Sichuan-Xizang Traditional Medicinal Plant, Chengdu University, Chengdu 610106, China; 3 School of Food and Biological Engineering, Chengdu University, Chengdu 610106, China

**Keywords:** ethylene response factors, growth-defense trade-off, transcriptional regulation, stress resilience, crop improvement

## Abstract

As sessile organisms, plants must dynamically allocate resources between growth and stress resilience. This review focuses on Ethylene Response Factor (ERF) transcription factors as central regulators of this fundamental balance. We evaluate the molecular basis of ERF function, highlighting their modular structure, dynamic post-translational regulation, and ability to form context-specific protein complexes that integrate diverse signals. While ERF family members show functional redundancy, certain ERF subgroups, such as the ERF-VIIs, exhibit clearer evidence of dual roles in coordinating both developmental programs and adaptive responses to stress. We further elucidate the mechanisms underlying ERF-mediated trade-offs, explaining how these factors direct spatial resource allocation and enable temporal switching between growth and defense states. Finally, we explore how emerging technologies, such as CRISPR-based genome editing and various synthetic biology tools, can harness ERF regulatory networks. These approaches offer promising strategies for engineering crops with precisely tuned adaptive capacity, supporting sustainable agriculture even in changing climate conditions. This synthesis highlights specific ERF subgroups as pivotal integrators and future targets for crop improvement.

## 1. Introduction

Plants are sessile organisms that must complete their life cycle under continuous exposure to changing and often unfavorable environments. To survive and reproduce, they need to resolve a fundamental dilemma: plants must allocate substantial resources to growth, organ formation, and reproduction, but they must also respond effectively to environmental stresses such as drought, flooding, salinity, temperature extremes, and pathogens. Because resources are limited, investment in adaptive stress responses frequently comes at the expense of growth and yield, whereas prioritizing growth can increase vulnerability to environmental damage [1,2]. How plants balance these competing demands represents a central focus of plant biology research.

This balance is inherently dynamic and context-dependent. Plants must adjust their priorities according to developmental stage, tissue identity, and stress intensity. Early developmental stages may favor rapid growth, while reproduction or survival may be more important in later stages [3]. Mild stress can trigger adaptive responses with limited impacts on growth, whereas severe stress often requires temporary growth suppression to ensure survival [4]. Importantly, plants must also resume normal growth once stress conditions are alleviated [5]. These shifting demands indicate that growth-stress coordination relies on flexible regulatory mechanisms rather than fixed developmental programs.

Transcription factors play crucial roles in mediating this flexibility [6,7]. Acting downstream of signaling pathways, they integrate hormonal and environmental inputs and control large sets of target genes [6]. Among plant transcription factor families, Ethylene Response Factors (ERFs) represent a particularly informative system for studying growth-stress coordination [8]. These transcription factors belong to the APETALA2/Ethylene Response Factor (AP2/ERF) superfamily and were initially characterized as terminal regulators of ethylene signaling. Ethylene is widely known for its roles in stress responses, senescence, and fruit ripening [9], but accumulating evidence shows that ERFs are also deeply involved in fundamental developmental processes, including root system architecture and vascular development, organ abscission, and the regulation of developmental phase transitions [8,10].

The prominent involvement of ERFs in both stress adaptation and development places them at a strategic point within plant regulatory networks. As major transcriptional outputs of ethylene signaling, ERFs interact extensively with other hormonal pathways such as those associated with jasmonic acid, abscisic acid, gibberellins, and auxin [8,10]. Through these interactions, ERFs can integrate multiple endogenous and environmental cues and generate context-specific transcriptional responses. Notably, individual ERF proteins can promote growth under favorable conditions while activating defense programs under stress, highlighting their broad functional versatility [11].

This versatility is enabled by the high molecular plasticity of ERF transcription factors. The activity of ERFs is regulated at multiple levels, including via transcriptional control, post-translational modification, protein stability, subcellular localization, and selective interactions with other transcription factors and/or co-regulators [12]. Consequently, ERFs function not as simple on-off switches but as adaptable regulatory platforms with outputs mediated by cellular and environmental context. In this review, we propose that ERF transcription factors act as key molecular balancers that dynamically coordinate plant development and adaptive stress responses, with substantial empirical support from the literature. We first summarize the molecular features that confer regulatory flexibility to ERFs. We then examine their roles in major developmental processes and stress responses. Finally, we analyze the mechanisms by which ERFs mediate growth-stress trade-offs and discuss how this knowledge may guide the development of improved crops that are not only resilient, but also productive, under fluctuating environmental conditions. What distinguishes this review from previous summaries is its integrative focus on the molecular plasticity underlying ERF function, spanning post-translational regulation, redox sensitivity, and context-dependent protein interactions, and its explicit connection of these mechanisms to the growth-defense trade-off framework. By synthesizing recent advances in ERF biology with critical evaluation of evidence gaps and translational challenges, we aim to provide not only a comprehensive overview but also a roadmap for future research and crop improvement strategies.

## 2. ERF Transcription Factors as a Molecular Toolbox: Plasticity and Regulatory Potential

### 2.1. Structural Basis and Functional Diversification

The ERF family is a large and widely distributed transcription factor family across the plant kingdom. In model plants and crops, the family comprises dozens to hundreds of members. For instance, *Arabidopsis* (*Arabidopsis thaliana* (L.) Heynh.) contains 122 *ERF* genes, rice (*Oryza sativa* L.) 131, maize (*Zea mays* L.) 166, and soybean (*Glycine max* (L.) Merr.) 323 [11]. Based on comprehensive phylogenetic analysis, the ERF family in different species can be divided into multiple distinct groups. Nakano et al. (2006) classified the *Arabidopsis* ERF family into 12 groups and the rice ERF family into 15 groups, with 11 of these groups being shared between the two species, indicating that the major functional diversification within the ERF family predated the monocot/dicot divergence [13]. Some groups, however, are species-specific, reflecting lineage-specific expansion and functional adaptation. Across all these groups, members are unified by the presence of a highly conserved DNA-binding domain.

This domain, known as the AP2/ERF domain, consists of approximately 60 to 70 amino acids and defines the ERF family [13]. This domain mediates sequence-specific binding to *cis* elements, such as the GCC box and related motifs, in the promoters of target genes [14]. Although the AP2/ERF domain is highly conserved across ERF family members, regions outside this domain can show substantial sequence divergence. Moreover, subtle variations within the AP2/ERF domain itself can create gradients of DNA-binding specificity, enabling ERF proteins to recognize a range of related but distinct DNA elements with varying affinities [8,10,12]. These binding specificity gradients, rather than discrete on/off target sets, allow closely related ERFs to differentially regulate overlapping sets of target genes, contributing to functional diversification within the family.

Many ERFs contain transcriptional activation domains enriched in acidic or glutamine-rich residues, which enable them to directly activate gene expression [12,15]. Others harbor repression domains, most notably the EAR motif, which recruits transcriptional co-repressors such as TOPLESS (TPL) and TOPLESS-RELATED (TPR) proteins [16]. Some ERFs even contain both activation and repression potential, suggesting that their regulatory effects can be dramatically mediated by their interacting molecular partners or cellular contexts [12]. For instance, ERF1 in *Arabidopsis* functions as a direct transcriptional activator of phytoalexin biosynthetic genes by integrating ethylene and jasmonate signals, while also serving as a phosphorylation substrate of mitogen-activated protein kinase 3/6 (MPK3/MPK6) kinases, which potentiates its activity [17]. This example illustrates that the functional output of an ERF is not fixed but dynamically modulated through multi-layered interactions, including synergistic cooperation with other transcription factors and signal-dependent post-translational modifications. This modular protein architecture allows ERFs to function as activators, repressors, or dual regulators, greatly expanding their functional range.

In addition to domain composition differences, subtle variation in the AP2/ERF domain itself can influence DNA binding affinity and specificity. These differences may contribute to selective target gene regulation among closely related ERF proteins [18,19]. Collectively, structural conservation combined with flexible regulatory domains provides a foundation for both functional redundancy and functional specialization within the ERF family.

### 2.2. Dynamic Transcriptional and Post-Translational Regulation

**Figure 1:**
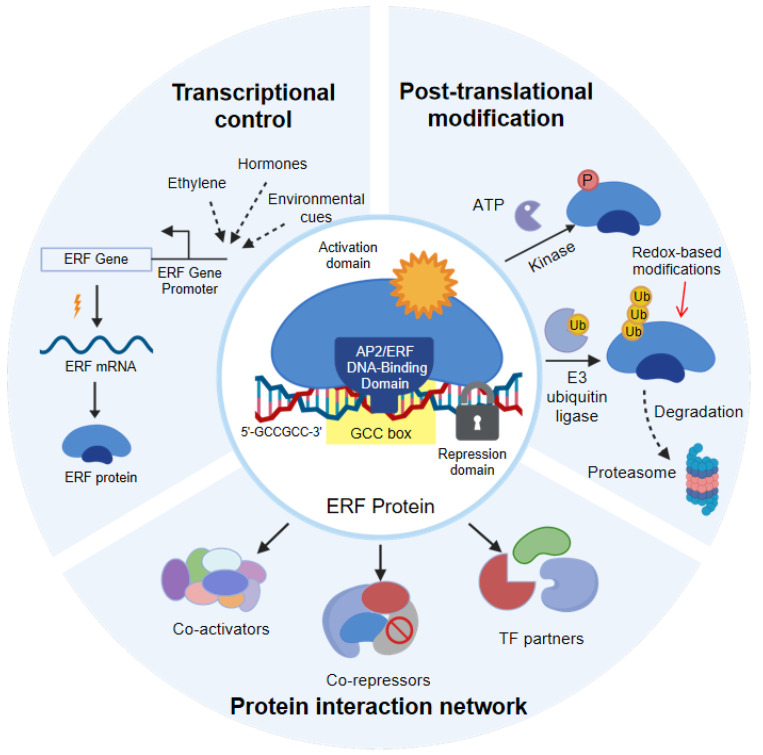
The ERF hub: a multilayer integrator of signals for plant adaptation.

In addition to having a distinctive structure, ERF proteins also exhibit activity that is strongly shaped by dynamic regulatory inputs (Figure 1). At the transcriptional level, *ERF* genes are themselves responsive to a broad range of signals. As their name indicates, ethylene is a major upstream regulator, but many *ERF* genes are also induced or repressed by jasmonic acid, abscisic acid, gibberellins, auxin, and environmental cues such as hypoxia, drought, salinity, and pathogen infection [8,10]. This responsiveness positions ERFs as early transcriptional responders that can relay hormonal and environmental information to downstream gene networks.

Post-translational regulation provides an additional layer of control that enables rapid and reversible modulation of ERF function [12]. Phosphorylation by stress-activated kinases can alter ERF DNA binding activity, transcriptional strength, or protein stability [20]. In several study systems, phosphorylation has been shown to enhance ERF activity under stress conditions, whereas dephosphorylation can promote attenuation of the response to the stimulus once the stress signal diminishes. For example, in *Arabidopsis*, pathogen-activated MPK3/MPK6 kinases phosphorylate AtERF6, which enhances its protein stability and transcriptional activity to boost defense gene expression [20]. Similarly, under osmotic stress, SnRK2s are activated and directly phosphorylate RaptorB, which leads to the dissociation of the target of rapamycin (TOR) complex and contributes to stress-induced growth inhibition [21]. Ubiquitination and subsequent proteasomal degradation also play key roles in controlling ERF abundance. For example, in *Arabidopsis*, the drought-responsive factor DREB2A is targeted for proteasomal degradation by DREB2A-interacting protein 1/2 (DRIP1/DRIP2) E3 ligases under non-stress conditions [22], while the light- and ethylene-signaling integrator RAP2.4 is destabilized by the constitutively photomorphogenic 1 (COP1) E3 ligase in the dark [23]. By regulating protein turnover, plants can fine-tune both the intensity and duration of ERF-mediated transcriptional responses. Other post-translational mechanisms, including changes in subcellular localization, further contribute to ERF plasticity. For example, some ERFs are retained in the cytoplasm under normal conditions and translocate to the nucleus only after stress or hormonal stimulation [8]. A recent study delineated a precise signaling cascade in which hypoxia-induced calcium signals are able to trigger the rapid activation and cytoplasm-to-nucleus translocation of the kinase calcium-dependent protein kinase 12 (CPK12) [24]. Upon entering the nucleus, CPK12 phosphorylates ERF-VII transcription factors, inhibiting their degradation via the N-end rule pathway, thereby stabilizing them for transcriptional activation. Such regulatory mechanisms enable plants to maintain ERFs in a primed yet inactive state, ready for rapid deployment as soon as conditions change.

Redox-based modifications provide an additional layer of ERF regulation under oxidative stress conditions [25]. For instance, the *Arabidopsis* ERF Rap2.4a undergoes redox-dependent conformational changes that modulate its DNA-binding activity: strongly reducing or oxidizing conditions impede promoter binding, while intermediate redox potentials promote binding and transcriptional activation [26]. This mechanism involves cysteine modifications that alter the protein’s oligomerization state. Similarly, the oxygen-dependent degradation of ERF-VII transcription factors via the N-end rule pathway represents another form of redox regulation, where the conserved N-terminal cysteine, acting as an oxygen sensor, regulates protein stability in response to fluctuating oxygen levels and redox conditions [27,28]. These redox-sensitive mechanisms allow ERFs to integrate cellular redox status with stress-responsive gene expression.

Together, transcriptional and post-translational controls enable ERFs to respond swiftly to fluctuating environments while limiting their prolonged or inappropriate activation and the associated costs.

### 2.3. Network Assembly Through Protein Interactions and Regulatory Complexes

The ERFs rarely act in isolation but rather function within complex regulatory networks formed through protein–protein interactions [29]. A defining feature of these networks is the ability of ERFs to physically interact with members of other transcription factor families, such as myeloblastosis (MYB) and NAM, ATAF1,2, CUC2 (NAC) proteins. This allows for regulation that goes beyond direct DNA binding. For instance, in loquat (*Eriobotrya japonica* (Thunb.) Lindl.), the ERF protein EjAP2-1, which cannot directly bind to its target promoter in this example, recruits MYB factors to cooperatively repress lignin biosynthesis genes [30]. Similarly, in tomato (*Solanum lycopersicum* L.) ripening, ERFs mediate ethylene signaling within a broader NAC-containing network to coordinate the ripening program [31]. Through such interactions, ERFs can modify DNA-binding specificity, alter transcriptional output, and impose combinatorial control of shared target genes, thereby extending their breadth of regulatory roles.

Interactions with co-regulators further expand ERF functionality. Repressive ERFs often recruit TPL or TPR co-repressors through their ERF-associated amphiphilic repression (EAR) motifs, thus leading to chromatin modification and stable gene repression [16]. This mechanism is exemplified in fruit ripening induced by ERFs such as FcERF12 in fig (*Ficus carica* L.) [32] and SlERF.F12 in tomato [33], which recruit TPL and histone deacetylases (HDACs) to form repression complexes that inhibit key ripening genes and delay softening. Conversely, ERF activation may be associated with transcriptional co-activators or components of the general transcription machinery. In *Arabidopsis*, activating ERFs can enhance transcription through cooperation with transcriptional co-activators or other regulatory proteins [34]. For example, the ERF transcription factor ORA59 recruits the mediator complex subunit 25 (MED25) to form a regulatory module that is essential for activating the expression of various defense-related genes and the biosynthesis of antimicrobial metabolites [35]. Collectively, these examples suggest that the recruitment of co-regulators by ERFs is a dynamic process that allows for flexible gene regulation in response to developmental cues and environmental changes. Importantly, competitive interactions among ERFs and other transcription factors can influence regulatory outcomes [29]. Closely related ERFs may exhibit competitive binding with respect to the same *cis* elements, while distinct ERFs may assemble alternative complexes on shared promoters. For instance, the tomato repressor ERF SlERF.F12 inhibits ripening by recruiting TPL2 and HDACs to form a repression complex that binds to key gene promoters [33]. Such competition and cooperation provide greater complexity of regulation that enables fine-scale tuning of gene expression. Thus, ERFs act as nodes within highly interconnected transcriptional networks rather than as linear signal transmitters.

### 2.4. Controversies and Unresolved Questions in ERF Biology

Despite significant advances, several controversies and unresolved questions remain in ERF biology. The strength of evidence for ERF functions varies considerably, with many conclusions derived from overexpression studies or heterologous systems that await confirmation under native conditions. Contradictory findings also remain underexplored. For instance, while ERF-VII members in *Arabidopsis* primarily activate core hypoxia-responsive genes [28], studies in rice have revealed that orthologous proteins can mediate distinct adaptive strategies, such as submergence 1A (SUB1A)-driven tolerance versus SNORKEL-mediated escape responses [27]. These functional differences, despite shared N-end rule regulation, highlight the importance of species-specific contexts. Yet systematic comparative analyses across species remain limited.

Alternative interpretations of existing data are often plausible. The apparent dual functionality of certain ERFs could reflect indirect effects mediated by downstream targets rather than intrinsic bifunctionality. For example, AtERF6 functions as a transcriptional activator upon phosphorylation [20], yet its broader regulatory effects on growth may involve additional factors not directly targeted by the protein itself. Moreover, context-dependent binding specificities observed in vitro may not fully capture the complexities of chromatin architecture and competing factors in vivo, as highlighted by studies on transcription factor interaction networks [29]. Addressing these gaps through meta-analyses, direct comparative studies across species, and more standardized experimental frameworks will be essential for moving the field toward a more robust understanding of ERF function.

As a whole, ERFs constitute a highly plastic regulatory toolbox in which a conserved DNA-binding core is coupled with flexible regulatory control (Figure 1). This design allows ERFs to integrate environmental and developmental signals and to generate context-dependent transcriptional outputs that coordinate plant growth and adaptive stress responses. However, several caveats should be considered when interpreting these findings. First, many protein–protein interactions and regulatory complexes have been characterized using heterologous systems or overexpression assays, and their physiological relevance under native conditions remains to be confirmed. Second, the functional significance of competitive binding among closely related ERFs, while mechanistically appealing, has been demonstrated in only a limited number of cases, and its genome-wide impact on gene expression awaits systematic investigation. Third, the extent to which the regulatory plasticity observed for individual ERFs (e.g., ERF1) reflects a general feature of the family versus exceptional cases remains unclear, as most family members lack detailed mechanistic characterization. Fourth, extensive redundancy within the ERF family complicates functional assignments, as loss-of-function phenotypes for individual ERFs are often masked by compensatory activities of paralogs. This raises questions about whether single ERFs act as decisive switches or whether regulatory outcomes emerge from network-level interactions buffered against individual perturbations. Addressing these gaps through native chromatin immunoprecipitation, tissue-specific perturbations, and comparative studies across diverse ERFs will be essential for moving from descriptive models to a predictive understanding of ERF-mediated transcriptional regulation.

## 3. Coordinating Development: Roles of ERFs at Key Stages of the Plant Life Cycle

### 3.1. Reproduction and Dispersal

**Figure 2:**
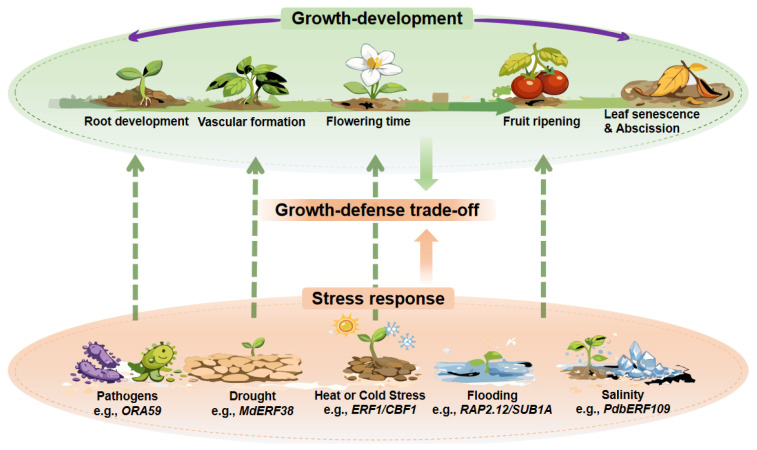
Coordination of development and stress responses by ERFs.

Reproductive success is critical to plant fitness, and ERF transcription factors play prominent roles in coordinating developmental programs that enable successful reproduction and seed dispersal [36] (Figure 2). Among the processes directly related to reproduction, fruit ripening and floral organ abscission represent two particularly well-studied examples in which ERFs act as core regulatory components tightly linked to ethylene signaling [37,38].

Fruit ripening is characterized by a burst of ethylene production that triggers extensive transcriptional reprogramming, leading to suites of changes in color, texture, flavor, and nutrient composition [39]. In climacteric fruits, such as tomato and banana (*Musa* spp.), ERFs function downstream of this ethylene surge to regulate ripening-related gene networks [40,41]. In tomato, the MADS box transcription factor ripening inhibitor (RIN) acts as a master regulator of ripening and directly or indirectly controls the expression of multiple *ERF* genes. These ERFs in turn regulate enzymes involved in cell wall modification, pigment accumulation, and ethylene biosynthesis, forming interconnected regulatory loops that ensure the coordinated progression of fruit ripening. For instance, the *rin* mutant fails to induce autocatalytic ethylene synthesis or achieve full fruit ripening, demonstrating that RIN (and ethylene acting through ERFs) is essential for gene expression associated with complete ripening [42]. Furthermore, the RIN-MC fusion protein responsible for the *rin* phenotype modulates hundreds of ripening genes, highlighting the scale of the RIN-centered regulatory networks that involve ERFs [43]. In bananas, ERFs such as MaERF11 modulate fruit ripening by acting as transcriptional repressors of ethylene biosynthesis and signaling genes. By fine-tuning the timing and intensity of ethylene responses, these ERFs contribute to the precise control of ripening onset and progression. For example, MaERF11 recruits the histone deacetylase MaHDA1 to repress genes involved in ethylene signaling and cell wall metabolism via histone deacetylation [44]. Additionally, MaERF11 itself is post-translationally attenuated by the ubiquitin E3 ligase MaRFA1, which weakens its repression of starch degradation genes [45]. Together, these examples highlight that ERFs do not merely execute ethylene signals but rather participate actively in shaping ripening dynamics through feedback and feedforward regulatory circuits.

In addition, ERFs play key roles in organ abscission, a developmental process essential for seed dispersal and the shedding of damaged or senescent organs. Abscission is tightly regulated by ethylene and involves the activation of cell separation programs acting in specialized abscission zones [38,46]. Several ERFs are specifically expressed in these zones and regulate genes that encode cell wall-modifying enzymes and signaling components. For example, tomato SlERF52 directly promotes the expression of cellulase and polygalacturonase genes to accelerate abscission [47]. Similarly, litchi (*Litchi chinensis* Sonn.) LcERF2 inhibits a UDP-glucose-4-epimerase gene (*LcUGE*), thereby reducing cell wall pectin content and enhancing the abscission of fruits [48]. Through these mechanisms, ERFs coordinate ethylene-dependent transcriptional responses that ensure abscission occurs at the appropriate developmental stage or in response to environmental cues.

### 3.2. Morphogenesis and Developmental Plasticity

The ERF family is deeply involved in determining plant architecture beyond reproductive structures and in enabling developmental plasticity in response to environmental conditions [12].

The ERF proteins contribute to root architectural responses to nutrient availability. Nitrogen and phosphorus deficiencies induce the expression of specific *ERF* genes that modulate lateral root formation and root hair development [34,49]. These responses can involve both synergistic activation and precise repression. For example, in apple (*Malus domestica* (Suckow) Borkh.), under drought stress, the ERF MdDREB2A coordinates nitrogen assimilation and root development by directly activating genes like *MdSWEET12*, among other genes, thereby optimizing growth under combined water and nutrient limitation [50]. Conversely, in physic nut (*Jatropha curcas* L.), the downregulation of *JcERF035* relieves the suppressive effect of phosphate deficiency on lateral root growth while promoting root hair formation [51]. Through such context-specific mechanisms, ERFs adjust root growth and branching to maximize nutrient acquisition. These developmental adjustments are deeply integrated with hormonal signaling pathways, particularly ethylene and auxin pathways, underscoring the integral role of ERFs as integrators of environmental cues and hormonal signals [34].

Additionally, ERFs have been implicated in vascular development and tissue patterning in plant aerial tissues [52]. For instance, in poplar (*Populus* spp.) wood formation, network analyses identified ERFs, including ERF118 and ERF119, as ethylene-signaling hubs that directly regulate xylem development [53]. Some ERFs regulate the expression of genes involved in xylem and phloem differentiation, thereby influencing transport capacity and mechanical support. Under drought stress, PtoERF15 in poplar enhances tolerance by directly activating the jasmonic acid signaling switch PtoMYC2b, which in turn regulates a suite of NAC transcription factors to optimize xylem vessel size and density for efficient water conduction [54]. Separately, in developmental contexts, PagERF81 from poplar 84 K (*Populus alba* × *P. glandulosa*) directly represses key lignin biosynthesis genes to modulate fiber cell wall thickness and lignification, thereby altering the mechanical strength of shoots [55]. Environmental factors, such as water availability and nutrient status, can modulate ERF activity, linking external conditions to adaptive responses in internal vascular development. For example, as detailed above, drought stress induces PtoERF15 to remodel xylem vessel development in poplar [54]. Through these functions, ERFs contribute to both the formation and the adaptive remodeling of plant structure.

### 3.3. Developmental Transitions and Senescence

Developmental phase transitions represent critical stages at which plants must reallocate resources and adjust physiological priorities [56]. Leaf senescence is a prominent example of such a transition, involving the controlled degradation of cellular components and the remobilization of nutrient resources to vegetative growth or reproductive tissues. Ethylene is a major hormonal regulator of senescence, and ERFs act as important mediators of ethylene-dependent transcriptional programs during this process [57].

Several ERFs are induced during leaf aging and regulate genes involved in chlorophyll degradation, protein breakdown, and nutrient transport. The precise timing of senescence is achieved through a balance between ERFs that promote and repress these processes. In *Arabidopsis*, the class II ERFs AtERF4 and AtERF8 promote senescence, as evidenced by premature aging under their overexpression and delayed aging in the double mutant [58]. In contrast, the tomato ERF SlERF.F5 represses leaf aging; its silencing accelerates senescence, suggesting it has a role in restraining the process, likely through modulating ethylene and jasmonic acid sensitivity [59]. By coordinating these opposing actions, ERFs ensure orderly senescence progression. Simultaneously, they integrate signals from other hormones, such as abscisic acid and jasmonic acid, which adjust the rate of senescence under stress [56].

Importantly, ERF-mediated regulation allows senescence to be adjusted in response to environmental cues [60]. This adaptability enables accelerated senescence under stress to facilitate the reallocation of resources for survival, while delayed senescence under favorable conditions can support growth. Thus, ERFs provide the crucial molecular link that translates external signals into the timing of developmental phase transitions.

### 3.4. Root System Remodeling Under Stress

Root system architecture exhibits remarkable plasticity in response to environmental stresses, and ERF transcription factors play key roles in mediating these adaptive changes. Under low oxygen conditions, ERF-VIIs, such as RAP2.2 and RAP2.12, are stabilized [28], which allows them to act as key regulators of the expression of hypoxia-responsive genes. These ERFs orchestrate both metabolic acclimation and developmental reprogramming to enhance plant survival. For instance, they can directly promote adaptive changes in root architecture; a recent study in *Arabidopsis* showed that the ERF-VII factor RAP2.12 promotes lateral root formation under hypoxia by upregulating abscisic acid-degrading enzymes, thereby fine-tuning hormone homeostasis to appropriately reshape the root system [61]. Beyond lateral roots, ERF-VIIs also mediate a shift in root system positioning by promoting the elongation of adventitious roots under waterlogged conditions, thereby strengthening the root system in oxygen-richer soil layers [62]. Through such mechanisms, hypoxia-regulated ERFs influence diverse root growth patterns, including adventitious root formation and altered elongation, enabling plants to realize adaptive responses to waterlogged environments.

In more severe flooding scenarios, ERF-VIIs govern strategic survival decisions that also impact root system function. In rice, this fundamental decision is governed by ERF-VIIs. The escape strategy is executed by ERFs, such as SNORKEL1 (SK1) and SNORKEL2 (SK2), which trigger a robust elongation response via gibberellin signaling [63]. Conversely, the tolerance strategy can be orchestrated by SUB1A, a well-understood ERF that restrains growth and reprograms metabolism to enhance survival during prolonged submergence [64]. Thus, through antagonistic regulation by distinct ERFs, plants can dynamically switch between escape and tolerance responses to flooding. This continuum of responses, ranging from developmental remodeling of root architecture to strategic whole-plant adaptation, demonstrates how the same family of transcription factors integrates environmental signals to optimize performance across a range of oxygen availability.

Overall, ERFs play roles at key developmental checkpoints throughout the plant life cycle. Acting downstream of ethylene and other hormonal signals, they coordinate transcriptional programs controlling fruit ripening, organ abscission, developmental transitions, and environmentally responsive morphogenesis. Through such roles in both structural development and developmental plasticity, ERFs link hormonal and environmental cues to the timing and spatial execution of growth and reproduction. However, the developmental functions described above should be interpreted with appropriate caution. First, many of the regulatory relationships have been established through gain- or loss-of-function studies that may amplify or mask context-dependent activities. Second, the extent to which individual ERFs contribute to multiple developmental processes versus specialized roles remains poorly defined for most family members. Third, the mechanistic understanding of how ERFs integrate into broader developmental networks is often inferred from phenotypic correlations rather than direct molecular evidence. Advancing beyond descriptive models will require spatiotemporally resolved analyses, conditional perturbation systems, and systematic mapping of ERF interactions within native developmental contexts.

## 4. Commanding Stress Adaptation: Multidimensional Defense Strategies Driven by ERFs

### 4.1. Metabolic Reprogramming and Osmoprotection

One of the primary ways in which plants respond to environmental stress is through modulating their metabolic pathways to maintain cellular integrity under adverse conditions [65]. In response to water stress, for example, plants accumulate compatible solutes, such as proline, trehalose, and flavonoids, which help to stabilize proteins and cellular structures, protect membranes, and maintain osmotic balance [66]. The ERF family plays an important role in regulating the expression of genes involved in the synthesis of these osmolytes, thus enhancing stress tolerance in plants. For instance, by regulating the biosynthesis of proline and other osmoprotectants, the apple ERF MdERF38 enhances cellular water retention, thus increasing drought resistance [67]. Similarly, other ERFs, such as those involved in flavonoid biosynthesis, help mitigate oxidative stress by promoting the accumulation of antioxidants. One compelling example is the tomato ERF SlERF7, which, upon UV-C-induced oxidative stress, directly binds to and activates the promoter of the key phenylpropanoid gene *SlPAL5*, leading to a pronounced accumulation of phenolic antioxidants [68]. Thus, ERFs act as central hubs in the metabolic reprogramming of plants under abiotic stress, supporting cell survival and function under unfavorable conditions.

### 4.2. Regulation of Ion and Redox Homeostasis

Maintaining ion homeostasis and managing oxidative stress are key aspects of plant responses to various environmental challenges, including salinity, drought, and pathogen infection [69]. The ERF family contributes to this homeostasis by regulating both ion transporters and antioxidant systems. For example, in hybrid poplar (*Populus davidiana* × *P. bolleana*), salt stress can induce the expression of *PdbERF109*. Overexpression of this gene enhances salt tolerance by directly boosting the activity of key antioxidant enzymes (e.g., superoxide dismutase, peroxidase, catalase), thereby strengthening reactive oxygen species scavenging and reducing oxidative damage [70]. The regulation of ion transporters by ERFs is vividly illustrated by cases in which their misexpression disrupts ionic balance. For example, in rice, overexpression of the ERF gene *OsERF106MZ* leads to the downregulation of the sodium transporter gene *OsHKT1.3*, thus contributing to increased salt sensitivity [71]. Similarly, overexpression of another rice ERF, *OsERF922*, results in a significantly increased shoot Na^+^/K^+^ ratio and compromised salt tolerance [72]. These examples underscore the importance of precise control of ERF in order to maintain ion homeostasis. Through such context-dependent activation or repression of both ion transporters and antioxidant components, ERFs ensure that plants can effectively manage the dual threats of ion imbalance and oxidative stress.

### 4.3. Systemic Signaling and Escape Responses

Beyond simple stress tolerance, plants can employ adaptive response strategies that enable them to withstand adverse conditions through developmental plasticity [73]. A classic example is provided by a typical response to flooding, in which plants face a critical choice between two contrasting survival strategies: an escape strategy involving rapid shoot elongation to regain air contact or a tolerance strategy involving metabolic arrest to conserve energy under water [74]. These contrasting strategies, mediated by ERF-VIIs such as SNORKEL1/2 and SUB1A in rice [63,64], exemplify how ERFs act as master interpreters of stress intensity and duration, guiding critical developmental decisions that balance immediate survival with long-term fitness in fluctuating environments.

Collectively, ERFs function as molecular arbiters of stress resilience. By dynamically tailoring transcriptional programs that fine-tune metabolism, ion transport, and growth, they empower plants with the critical ability to strategically choose between stress tolerance and developmental escape, ultimately enabling their survival in even fluctuating environments. However, several limitations should be acknowledged. First, most studies have examined ERF functions under single stress conditions, leaving their roles under combined stresses typical of field environments largely unexplored. Second, the relative contribution of ERFs compared to other stress-responsive families such as NACs and MYBs remains poorly quantified. Third, translational validation in diverse crop backgrounds under agronomic conditions is still limited. Addressing these gaps will be essential for leveraging ERF-based strategies in practical crop improvement.

## 5. Molecular Mechanisms of Growth-Defense Coordination by ERFs

### 5.1. Signal Integration and the Modulation of ERF Regulatory Output

**Figure 3:**
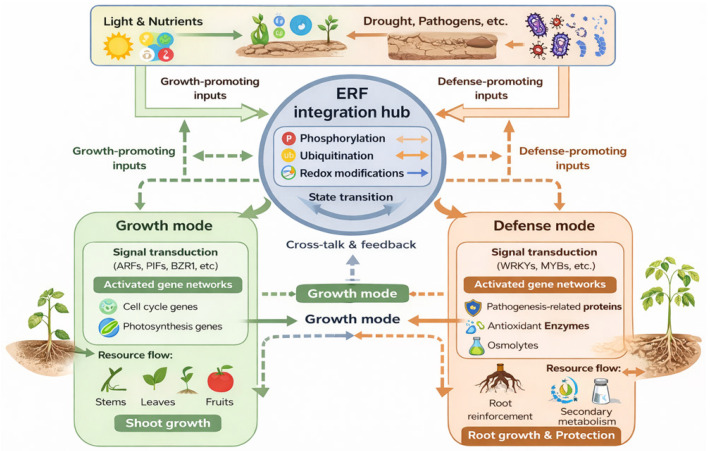
Ethylene response factor-mediated resource allocation under stress.

A key feature of ERFs is their ability to integrate multiple signaling pathways and respond to different environmental cues in an appropriate context-specific manner [8] (Figure 3). To achieve this, individual ERF members undergo precise, condition-dependent regulation. For instance, in *Arabidopsis*, AtERF6 is phosphorylated and stabilized upon pathogen attack to potentiate defense responses [20], while the stability of AtERF74/RAP2.4 is modulated by both light and ethylene signals [23]. This regulatory plasticity enables a single ERF to function in counterposed regulatory mechanisms. Under favorable environmental conditions, certain ERFs can promote growth, whereas under stress, they swiftly reprogram transcription to activate defense mechanisms, a pivotal function that establishes ERFs as central hubs in balancing growth and adaptive stress responses [8,12]. However, direct evidence for most ERFs simultaneously regulating both growth and defense pathways in the same cells remains limited and requires further investigation.

This functional shift is mediated by multiple layers of regulation. Upstream signals from different hormones, such as abscisic acid under drought or salicylic acid during pathogen attack, can alter the activation of *ERF* genes [75,76]. Mechanistically, abscisic acid promotes drought tolerance in tomato by triggering the degradation of the repressor ERF.D2, thereby promoting jasmonic acid biosynthesis [77]. Similarly, salicylic acid fine-tunes immune signaling by inducing a set of ERF transcriptional repressors that modulate the activity of key activators such as ORA59 [78]. Post-translational modifications, including phosphorylation and ubiquitination, further fine-tune the activity of these ERFs, thus affecting their stability, subcellular localization, and transcriptional activity [20,22,24]. Additionally, selective protein–protein interactions can form distinct transcriptional complexes, allowing ERFs to activate or repress specific sets of genes. This is exemplified by ERFs recruiting co-repressors like TOPLESS to inhibit gene expression [33] or by partnering with other transcription factors (e.g., MYBs) to realize combinatorial control [30]. Such interactions are fundamental features of ERF-mediated regulatory networks [29]. This regulatory plasticity allows various ERFs to promote developmental processes under optimal conditions and rapidly switch to activating stress-response pathways under adverse environments, enabling their role as core integrators of plant growth and adaptive stress responses.

### 5.2. Spatial and Temporal Regulation of the Growth-Defense Trade-Off

The core integrative function of ERFs is perhaps most vividly exhibited in their ability to manage the fundamental trade-off between growth and defense. Plants must continually balance growth and defense, especially when faced with environmental stress [1,2,4]. It is important to note, however, that ERFs operate within a broader regulatory network that includes other central hubs, such as DELLA proteins and TOR kinase, which also exert control over resource allocation in response to environmental cues [79,80]. One of the ways in which ERFs play roles in maintaining this balance is by mediating spatial and temporal trade-offs in resource allocation [1].

#### 5.2.1. Spatial Trade-Offs: Resource Allocation Among Tissues

Under stress conditions, particularly drought, ERFs coordinate plant responses by prioritizing resources for critical tissues, such as the root system, while limiting growth in other, perhaps less crucial areas. This spatial reallocation is driven by stress hormones that include ethylene and abscisic acid, which redirect growth and metabolic resources under water deficit conditions [9]. At the molecular level, ERFs execute this trade-off by integrating key hormonal signals—such as ethylene (which influences root development) and auxin (which modulates both root and shoot growth)—and translating them into appropriate transcriptional reprogramming [8,9]. Thus, they activate root-supporting genes while repressing shoot-growth programs, thereby mediating the systemic metabolic adjustments characteristic of adaptive responses to drought [9,66].

This mechanistic framework is illustrated by specific ERF functions. For example, during drought, the apple ERF MdDREB2A coordinates nitrogen assimilation and root development by directly activating genes such as *MdNIR1* and *MdSWEET12*, thereby optimizing growth under combined water and nutrient limitation [50]. Conversely, ERF transcriptional repressors, such as *Arabidopsis* AtERF4, can accelerate leaf senescence, a process that halts aerial growth and remobilizes resources, thereby conserving both water and energy [58].

#### 5.2.2. Temporal Trade-Offs: Sequential Prioritization of Growth and Defense

In addition to spatial trade-offs, ERFs also mediate temporal trade-offs by regulating growth priorities variably throughout time [1]. This dynamic control enables ERFs to function as molecular switches, rapidly toggling between growth and defense prioritization. Under acute stress, such as rapid drought or pathogen attack, ERFs coordinate the rapid suppression of growth to prioritize defense. A classic example of this phenomenon is illustrated by *Arabidopsis ERF1*, which, when overexpressed, constitutively activates a broad suite of defense genes while concurrently suppressing growth, ultimately leading to a stunted phenotype. This demonstrates the role of ERFs in initiating the temporal shift from growth to defense [81]. However, such switches can be reversible, ensuring growth can resume upon cessation of stress conditions. A key mechanism for this temporal reset is the oxygen-dependent degradation of specific ERFs. The ERF-VIIs, e.g., RAP2.12, are stabilized under hypoxia to activate adaptive responses but are rapidly targeted for proteasomal degradation via the N-end rule pathway upon reoxygenation [28]. This degradation is precisely controlled by plant cysteine oxidases, which act as oxygen sensors that initiate this process, serving as a molecular timer that terminates the hypoxia response and allows for the subsequent resumption of growth [82].

Thus, by orchestrating mechanisms from transcriptional reprogramming to stimulus-triggered protein turnover, ERFs can act as a time-sensitive transcriptional rheostat, enabling plants to sequentially prioritize survival and, upon stress relief, efficiently resume growth.

### 5.3. Context-Dependent Assembly of ERF Regulatory Networks

Another aspect of how ERFs coordinate growth and defense is demonstrated by their roles in regulatory modules. Rather than acting in isolation, ERFs act as parts of larger regulatory networks that integrate various signals to produce specific functional outputs [8]. This modular organization of transcription factors is a fundamental element of generating specific biological responses in plants [83]. This context-dependent assembly is exemplified by ERFs engaging with specific hormonal signaling pathways. Under favorable growth conditions, ERFs can interact with components of the gibberellin signaling pathway to nucleate transcriptional complexes that promote cell division, elongation, and other growth-related processes [84]. Conversely, under biotic or abiotic stress, different ERFs can preferentially associate with factors in the jasmonic acid signaling pathway, thus forming complexes that activate the expression of defense-related genes, such as those encoding pathogenesis-related proteins and antioxidant enzymes [85].

The ability of ERFs to participate in alternative protein complexes, thereby committing their associated transcriptional machinery to either growth or defense programs, represents a sophisticated mechanism of cell-level decision-making [83]. This model aligns with the broader understanding of transcription factors acting as dynamic integrators within flexible regulatory networks, in which signal-dependent protein–protein interactions can determine functional output [86]. However, direct evidence for individual ERFs switching between growth and defense complexes remains limited, as most studies have characterized different ERFs in specific contexts rather than tracking the same protein across conditions. Whether this plasticity occurs at the level of individual ERFs or reflects family-wide functional diversification awaits further investigation. Nonetheless, the existing evidence firmly establishes that ERFs as a family exhibit context-dependent assembly, enabling precise switching between physiological states to optimize resource allocation.

In summary, ERFs are central regulators of the growth-defense tradeoff in plants. They achieve this by integrating multiple signals, directing resource allocation response to variation occurring in both space and time, and forming condition-specific regulatory complexes. This allows plants to dynamically adjust their priorities between growth and defense in response to changes in environmental conditions, thereby enhancing their fitness. However, several limitations should be acknowledged when interpreting these models. First, most evidence for spatial and temporal trade-offs derives from correlative observations or overexpression phenotypes, with direct demonstration of ERF-mediated resource reallocation under native conditions still lacking. Second, while the conceptual framework of context-dependent assembly is compelling, it rests largely on examples of different ERFs in different contexts; whether individual ERFs dynamically switch partners remains to be rigorously tested. Third, the quantitative contribution of ERF-mediated mechanisms relative to other regulatory layers (e.g., hormone signaling, metabolic flux) has not been systematically assessed. Addressing these gaps through live imaging, quantitative proteomics, and systems-level approaches will be essential for transforming descriptive models into a predictive understanding of growth-defense coordination.

## 6. Engineering ERF Networks for Next-Generation Crops

**Figure 4:**
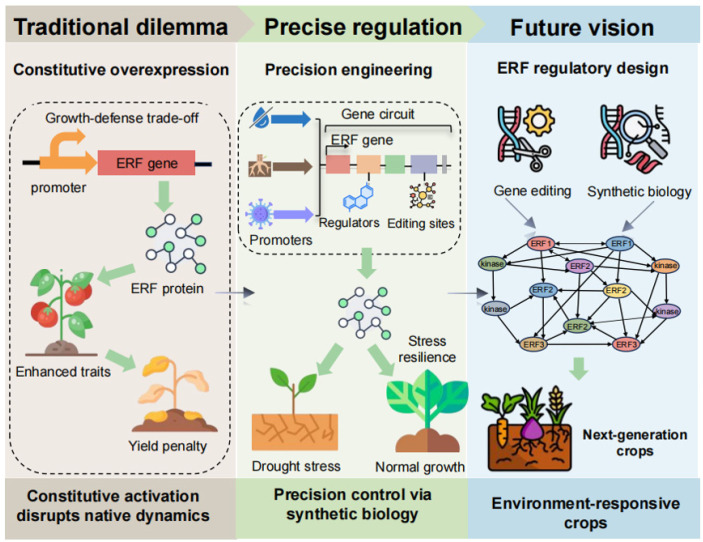
Strategies for engineering crops with a focus on ERFs.

The pursuit of crop resilience through genetic improvement has logically targeted central gene regulators, including ERFs. The future strategies for engineering these networks are summarized in Figure 4. Conventional transgenic strategies, often involving constitutive overexpression of *ERF* genes, have validated their potential for enhancing tolerance to drought, salinity, and pathogens. However, these gains are frequently offset by a substantial cost in terms of agronomic performance, including reduced biomass, stunted growth, and lower yields [87]. This trade-off directly reflects the core biological role of ERFs as master regulators of growth-defense balance. Their activation disrupts this equilibrium, however, demonstrating that the future of ERF-based crop improvement depends on the precise orchestration of this system rather than simply overriding it.

This need has motivated the development of more refined crop improvement strategies. The utilization of tissue-specific or stress-inducible promoters offers a path toward contextual control achieved by restricting ERF activity to the tissues and times of actual need [88,89]. However, even such targeted approaches carry pleiotropic risks, as ERFs often participate in multiple regulatory networks and their manipulation may inadvertently affect unrelated developmental pathways [34]. This underscores the need for comprehensive phenotypic analysis when engineering ERF activity to anticipate potential off-target effects. More specifically, CRISPR-based genome editing now enables direct and fine-scale modification of ERF networks themselves. Editing promoter regions to modulate expression, altering protein-interaction domains to reprogram signaling outputs, or modifying stability-determining residues can advance crop engineering beyond simple implementations of gene overexpression [90,91]. However, the extensive functional redundancy within the ERF family means that editing single loci may yield minimal phenotypic effects, highlighting the need for multiplex editing strategies to simultaneously target multiple family members or their shared regulatory elements to overcome this challenge in engineering complex polygenic traits [80]. The ultimate goal is to engineer allelic variants that recalibrate the stress response threshold, thereby enhancing resilience only as needed and avoiding the growth costs associated with their constitutive expression.

Fully realizing this potential requires addressing several interconnected challenges. A foremost frontier is deciphering ERF network plasticity under combined stress conditions. In the field, plants face co-occurring stresses, such as drought, heat, and pathogens. These stresses trigger distinct yet interconnected signaling pathways, and ERFs often sit at nodes where these pathways converge. The outcome of ERF activation under combined stresses is not simply additive but reflects complex crosstalk that can result in synergistic or antagonistic interactions. For instance, drought and heat stress can activate opposing hormonal responses, with ERFs prioritizing one pathway over another depending on stress intensity or duration. Deciphering these hierarchical prioritization mechanisms, specifically how plants weigh and integrate conflicting signals, will be critical for predicting ERF network behavior under real-world conditions. Understanding how ERF-mediated signaling hierarchies integrate and adapt under such multi-stress scenarios is essential for engineering robust, real-world resilience [92]. Emerging single-cell and spatial genomics technologies will be vital research tools in this effort, providing the resolution needed to map ERF activity and function across specific cell types under dynamic stress conditions, thereby informing targeted engineering [93,94]. Such integrative approaches, powered by single-cell and spatial genomics, will allow us to dissect ERF networks with unprecedented cellular and spatial precision, charting a course for targeted engineering [95].

Ultimately, the most transformative opportunities may arise from synthetic biology [96]. Beyond adjusting existing components, the design of synthetic genetic circuits that utilize ERF pathways as programmable logic modules is particularly promising. Initial progress toward this vision has already been made. For instance, a synthetic MAMP-responsive promoter integrating both activating and repressing ERFs has been constructed, demonstrating a functional ERF-based regulatory module [97]. More recently, manipulating specific ERFs has been shown to modulate flavonoid biosynthesis in citrus, providing an example of ERF-based metabolic engineering for valuable natural products [98]. These circuits could integrate multiple environmental sensors to drive dynamic, context-specific, and reversible stress responses, optimizing resource allocation in real time. However, significant challenges remain, including network complexity, cross-talk with endogenous pathways, and the need for precise spatio-temporal control. Moreover, translating these proof-of-concept studies into field applications will require overcoming additional hurdles, including circuit stability across generations, potential fitness costs, and regulatory constraints for genome-edited crops. Importantly, ERF-based strategies do not always yield desired outcomes. Manipulation of ERF activity has been associated with developmental penalties such as stunted growth or reduced fertility, reflecting their pleiotropic roles. Moreover, gains in stress tolerance have sometimes proven transient or context-dependent, failing to confer durable resilience. These examples underscore the need for rigorous field testing and a deeper understanding of ERF regulatory dynamics. This approach would mark a paradigm shift: from engineering static traits to programming adaptive capacity. This offers a promising path to equip crops with the built-in resilience necessary to remain productive under increasingly variable and unpredictable climates. The path from understanding ERFs as molecular mechanisms underlying homeostasis to programming them as master regulators of the architecture of crop adaptation is now clearly defined.

## 7. Conclusions

The ERF transcription factors are central to one of the most critical equilibria in biology: the dynamic balance between plant growth and environmental resilience. This review has outlined how ERFs, as master regulators of plasticity, function not as simple switches but as sophisticated integrators (see Table 1 for representative studies across diverse species). However, ERFs do not act alone. Other transcription factor families, including NAC, WRKY, and bZIP, also play key roles in stress and developmental signaling. Whether ERFs are uniquely central or function within a distributed network of partially redundant regulators remains an open question. While direct evidence for most ERFs simultaneously regulating both growth and defense pathways in the same cells remains limited, the regulatory plasticity observed across the family underscores their potential to coordinate these processes. They convert combined hormonal and environmental signals into precise transcriptional programs, allowing plants to prioritize growth under favorable conditions and pivot to defense when faced with adverse conditions. This molecular flexibility, achieved through layered regulation and dynamic network interactions, underpins physiological adaptability.

Their mechanistic role is pivotal: environmental cues converge on ERF hubs, triggering a reconfiguration of ERF activity, stability, and partnerships within transcriptional complexes. This reshaped regulatory state can then drive context-specific transcriptional output, allocating resources appropriately to either growth or stress adaptation. Through this finely tuned conditional logic, ERFs regulate the growth-defense trade-off, enabling plants to maintain fitness despite variable environmental conditions.

The future of crop improvement hinges on moving from merely discovering and characterizing ERF functions to actively utilizing their regulatory logic in genetic engineering. To develop crops that are both productive and resilient, we must advance beyond conventional overexpression-based approaches. This entails precisely modulating ERF expression with spatiotemporal control, editing their regulatory architecture, and programming the capacity for adaptive responses through synthetic biological circuits. By harnessing the balancing principles of ERFs, we can design next-generation crops endowed with innate yet dynamic resilience to thrive in an increasingly unpredictable climate. The path from fundamental insight to transformative application utilizing these versatile transcriptional balancers is now clearly charted.

## Figures and Tables

**Table 1:** Representative studies of ERF transcription factors across diverse plant species.

Species	ERF Gene	Function	Experimental Approach	Ref
*Arabidopsis*	*AtERF6*	Defense response	Phosphorylation assay, mutant analysis	[20]
	*PYL*s (e.g., *PYL1*)	ABA signaling/Growth recovery after stress	Phosphoproteomics, kinase assay, mutant analysis	[21]
	*RAP2.12*	Hypoxia response	N-end rule degradation, ChIP	[28]
	*ERF1*	Defense response	Overexpression, transcriptomics, ChIP	[17,81]
	*AtERF4*	Leaf senescence	Overexpression, mutant analysis	[58]
Rice	*SUB1A*	Submergence tolerance and drought tolerance	Overexpression, near-isogenic line analysis	[64]
	*SNORKEL1*/*2*	Deep water escape	Overexpression, hormone analysis	[63]
	*OsERF106MZ*	Salt sensitivity	Overexpression, ion measurement	[71]
	*OsERF922*	Salt sensitivity	Overexpression, ion analysis	[72]
Tomato	*SlERF.F12*	Fruit ripening repression	CRISPR/Cas9, ChIP-seq	[33]
	*SlERF52*	Flower abscission	Overexpression, expression analysis	[47]
	*SlERF7*	UV-C response, antioxidant	EMSA, transactivation	[68]
	*ERF.D2*	Drought sensitivity	Degradation assay	[77]
Apple	*MdDREB2A*	Drought response, N assimilation	Overexpression, ChIP-qPCR, physiological assays	[50]
	*MdERF38*	Drought-induced anthocyanin	Overexpression, metabolite analysis	[67]
Poplar	*PtoERF15*	Drought tolerance	Overexpression, transcriptomics	[54]
	*PagERF81*	Lignin biosynthesis repression	EMSA, gene editing	[55]
	*PdbERF109*	Salt tolerance	Overexpression, enzyme assays	[70]
Citrus	*CitERF32*/*33*	Flavonoid biosynthesis	DAP-seq, dual-luciferase	[98]
	*CitRAV1*	Flavonoid regulation	Protein interaction	[98]
Banana	*MaERF11*	Fruit ripening repression	Protein interaction assays, epigenetic analysis	[44]
	*MaRFA1*	Post-translational regulation	Ubiquitination assay	[45]
Loquat (*Eriobotrya japonica*)	*EjAP2*-*1*	Lignin repression	Protein interaction assay, transcriptional activity assay	[30]
Litchi (*Litchi chinensis*)	*LcERF2*	Fruit abscission	DNA-binding assay, transcriptional activity assay	[48]
Fig (*Ficus carica*)	*FcERF12*	Fruit ripening	Transcriptional repression assay, protein interaction analysis	[32]
Physic nut (*Jatropha curcas*)	*JcERF035*	Phosphate deficiency response	Gene expression analysis	[51]

## Data Availability

No new data were created or analyzed in this study. Data sharing is not applicable to this article.

## Abbreviations

The following abbreviations are used in this manuscript:

ERF	Ethylene Response Factor
AP2/ERF	APETALA2/Ethylene Response Factor
NAC	NAM, ATAF1,2, CUC2
MYB	Myeloblastosis
TPL	TOPLESS
TPR	TOPLESS-RELATED
MED25	Mediator complex subunit 25
MPK3/MPK6	Mitogen-activated Protein Kinase 3/6
SnRK2	Sucrose Non-fermenting 1-Related Protein Kinase 2
DRIP1/DRIP2	DREB2A-Interacting Protein 1/2
CPK12	Calcium-Dependent Protein Kinase 12
SK1/SK2	SNORKEL1/SNORKEL2
TOR	Target of rapamycin
HDAC	Histone Deacetylase
RIN	Ripening Inhibitor
SUB1A	Submergence 1A
CRISPR	Clustered Regularly Interspaced Short Palindromic Repeats
EAR	ERF-associated Amphiphilic Repression
COP1	Constitutively Photomorphogenic 1
